# Calendar

**DOI:** 10.1155/S1463924680000685

**Published:** 1980


### Photoacoustic spectrometer

EDT's photoacoustic spectrometer, model OAS 400, is now available with an IEEE 488 computer interface to which can be connected directly a wide variety of minicomputers and microcomputers. This allows the instrument to be controlled by the computer and for data processing and manipulation to take place inside the computer, prior to the completed spectrum being displayed to the integral xy recorder for hard copy storage.

A range of software programs for the Commodore PET microcomputer have been developed by EDT. These allow the user to acquire up to four spectra as well as a carbon black reference spectrum for subsequent manipulation and processing. Further spectra can be stored on a floppy disc which is an optional extra with the system. Two spectra can be added, subtracted or given as a ratio allowing baseline subtraction and normalisation of lamp intensity. User specific programmes can be developed using the optional BASIC compiler.

The program in standard form is designed to run on a 32K PET with cassette program storage. An alternative version designed for use with the Comp-u-Think diskette is also available.

*EDT Research, 14 Trading Estate Road, London NW10 7LU, UK.*

### Liquid scintillation counter

Microprocessor control and automatic analysis of up to 300 samples per run are features of Beckman's LS-7500 liquid scintillation counter. The counter has a built-in library of ten standard counting programs that satisfy the most frequently requested liquid scintillation applications. Command tower programming gives the operator the versatility to edit counting parameters.

Command towers, inserted in the conveyor belt preceding a group of samples, select any one of the 10 programs. The user sets a command tower and pushes the Auto button to activate the program. Time and error of the program is edited by dialling a change on the second command tower. A program summary is printed automatically to confirm the desired counting parameters. Up to 300 samples can then run unattended. Instrument calibration and quench monitoring by H number are also performed automatically.

*Beckman-RIIC Ltd, Turnpike Road, Cressex Industrial Estate, High Wycombe, Bucks HP12 3NR, UK.*

### Calendar

**Editor's Note:**
*Organisers of conferences, seminars etc. should send details for inclusion in this calendar as soon as the relevant information is available and not later than three months before the event.*

**Microcomputer applications in health care**
December 5, London.
*Dr J.P. Woodcock, Department of Medical Physics, Bristol General Hospital, Guinea Street, Bristol.*

**Atomic absorption and emission spectrometry**
December 8-12, Loughborough.
*Dr J.F. Tyson, Department of Chemistry, Loughborough University of Technology, Loughborough, LE11 3TU.*

**Salon du Laboratoire**
December 8-13, Paris.
*Commissariat General, 40 rue de Colisee, 75381 Paris cedex 08, France.*

**Laboratory methods of handling and analysing toxic substances**
December 9, London
*The Secretary, Analytical Division, The Chemical Society, Burlington House, London W1.*

**Microprocessors and microcomputers, interfacing and applications**
December 14-19, Tallahassee, Florida
*American Chemical Society, 1155 Sixteenth Street, N.W., Washington DC 20036, USA.*

### 1981

**London Laboratory Conference**
February 24-26, London.
*A.J. Holdsworth, Manager, Exhibition & Conference Group, Morgan Grampian (Process Press) Ltd, 30 Calderwood St, London SE18.*

**The use of chemical nomenclature**
March 24-26, London.
*The Symposium Organiser, Laboratory of the Government Chemist, Cornwall House, Stamford St, London SE1.*

**Laboratory '81**
April 1-2, Glasgow
*Curtis Steadman, 34-36 High Street, Saffron Waldon, Essex.*

**Biotechnology – second European Congress**
April 5-10, Eastbourne
*Secretariat, ECB2, c/o Society of Chemical Industry, 14 Belgrave Sq, London SW1 8PS. UK.*

**International symposium on electroanalysis in clinical, environmental and pharmaceutical chemistry.**
April 13-16, Cardiff.
*Short Courses Section (Electroanalysis Symposium), UWIST' Cardiff CF1 3NU.*

**Anglo-Dutch symposium on quantitative organic analysis**
April 23-24, Noordwijkerhout, Holland
*Miss P.E. Hutchinson, Royal Society of Chemistry, Analytical Division, Burlington House, London W1.*

**Association of Official Analytical Chemists, 6th Annual Spring Workshop**
May 12-14, Ottawa.
*Donald L. Grant, Health Protection Branch, Health and Welfare Canada, Tunney's Pasture, Ottawa, Ontario KIA 0L2, Canada.*

**Automatic chemical analysis summer school**
July 5-10 Swansea.
*Professor D. Betteridge, Department of Chemistry, University College of Swansea.*

**Euroanalysis IV**
August 23-28, Helsinki, Finland.
*Association of Finnish Chemical Societies, Executive Secretary, Pohj, Hesperiankatu 3B10, SF-oo260 Helsinki 26, Finland.*

### 1982

**12th Annual Symposium on the Analytical Chemistry of Pollutants**
April 14-16, Amsterdam.
*Prof Dr R.W. Frei, Congress Office, 12th Annual Symposium on the Analytical Chemistry of Pollutants, Congress Bureau, Vrije Universiteit, PO Box 7161, 1007 MC Amsterdam, The Netherlands.*

**International Congress on Automation in the Clinical Laboratory**
April 19-22, Barcelona, Spain.
*Dr. R. Galimany, Departmento de Analisis Clinicos, Seccion de Automatizacion, Cuidad Sanitaria 'Principes de Espana', Hospitalet de Llobrogat, Barcelona, Spain.*

